# Turnover behavior and intention among dentists and medical doctors: a cross-sectional study in China

**DOI:** 10.1186/s12903-024-03903-9

**Published:** 2024-02-04

**Authors:** Keying Shi, Yong Wang, Zhe Sun, Jing Zhao, Fangyue Xiang, Zhi Chen, Wenjing Sun, Yuanna Zheng

**Affiliations:** 1 School/Hospital of Stomatology, Zhejiang Chinese Medical University, Hangzhou, Zhejiang China; 2 Department of Stomatology, Shangcheng District Jiubao Community Health Center of Hangzhou City, Hangzhou, Zhejiang China; 3 Ningbo Dental Hospital/Ningbo Oral Health Research Institute, Ningbo, Zhejiang China

**Keywords:** Turnover, Intention, Medical doctors, Dentists, Working-related factors

## Abstract

**Background:**

Retention of doctors is a global challenge and doctors working in different departments may face different problems. The study aimed to explore the turnover behavior and intention and correlated factors among Chinese dentists and medical doctors in other clinical fields.

**Methods:**

A cross-sectional study was conducted online in 5 regions of China from March 12th to April 12th, 2020. The questionnaire included 3 parts, socio-demographic characteristics, turnover behavior and intention, and concerns about work-related factors. Chi-square test and/or Wilcoxon Mann-Whitney test were applied for comparison, and binary logistic regression was used for finding the factors.

**Results:**

A total of 2428 eligible questionnaire were received, comprising 1954 responses from dentists and 474 from medical doctors. Rates of turnover behavior among dentists and medical doctors were 2.87% and 6.96%, respectively. Similarly, rates of turnover intention were 51.79% among dentists and 71.20% among medical doctors. Educational level was negatively correlated with turnover behavior of both medical doctors and dentists, and concern about salary was a unique negatively correlated factor for dentists. Age was negatively correlated with turnover intention in both medical doctors and dentists. Conversely, concerns about workload and doctor-patient relationship were positively correlated with turnover intention in both groups. Concern about salary was the distinct correlated factor of medical doctors’ turnover intention, while gender and annual household income were correlated with turnover intention among dentists.

**Conclusions:**

Low turnover rate but high turnover intention rate was the current status of Chinese doctors’ employment. Turnover behavior and intention were more optimistic among dentists than medical doctors. Factors related to turnover behavior and turnover intention were not identical among dentists and medical doctors. Therefore, personalized retention measures were necessary for dentists and medical doctors.

## Background

The shortage of health workers is a worldwide topic. It is estimated that the world will face a shortage of up to 14 million health workers by 2030, of which about 2.3 million are doctors [1]. Attracting people to the medical industry and training them to be qualified health workers in order to reverse the situation takes a long time, especially for doctors. In this case, retention of doctors becomes important [2]. Castro Lopes et al. [3] conducted a rapid review of studies published since 2005 and revealed that the annual attrition rate of doctors in low-income countries and middle-income countries reached 15% and 9.8%, respectively. According to a survey in UK, the turnover rate of general practitioners attained over 14% in 2019 [4]. Besides turnover rate, leaving behavior can be effectively predicted by turnover intention, which refers to the predisposition to leave the organizations or careers [5]. A study showed that almost a half of the physicians in Switzerland had the thoughts of leaving the profession [6]. In Korea, 30.5% of 2719 surveyed doctors intended to leave the profession within 2 years [7]. Meanwhile, among 20,785 physicians working in tertiary hospitals across 31 provinces in China, 20.5% of them intended to change the career [8].

To identify factors that contribute to turnover is essential for developing effective strategies to retain doctors. Previous studies verified that work overload [6, 9, 10], unsatisfied income [7, 11], and workplace violence [12–14] were positively linked to turnover intention. Meanwhile, fearing of infecting diseases or a low level of confidence of safety measures in preventing disease infection played an important role on turnover intention [15, 16].

Population growth and aging have led to cumulative burden of oral condition in the world [17]. Most of the World Health Organization (WHO) member states reported to have less than 1 dentist per 1000 population in 2019 [18]. While China faced greater challenges with only 0.175 dentists per 1000 population in 2020, and the allocation of dentist resources was uneven [19, 20]. In a recent study evaluating the mental health of Chinese dentists, it was found that out of 1,855 participants, 25.7% reported experiencing psychological distress. Additionally, 28.9% of the Chinese dentists reported job burnout, and a notable 24% expressed regret regarding their career choice [21]. This result may have a negative impact on the retention of dentists. To date, studies on Chinese dentists’ turnover status have been limited.

At the same time, it is questionable whether the results of previous studies on the turnover status of the doctor population as a whole or doctors in fields other than dentistry, named as medical doctors for distinguish, can be directly applied to dentists. Many differences have been found in the working-related factors between dentists and medical doctors. In Taiwan and Brazil, medical doctors’ workload and income were higher than dentists’ [22, 23]. Workplace violence in dental department was less common than in other clinical departments [24]. A meta-analysis of global prevalence of occupational exposure to bloodborne pathogens among doctors revealed that the incidence of needlestick injuries in dentists was lower than that of surgeons [25]. Therefore, the aims of the present study were to investigate the turnover behavior and intention rate in both dentists and medical doctors in China, and further to identify potential factors related to the turnover behavior and intention.

## Subjects and methods

### Study design

The cross-sectional survey was conducted from March 12th to April 12th, 2020. One university with a medical profession and one medical university were randomly selected from the five regions, which were East China, North China, South China, Central China, and West China, and 10 universities were finally included. The self-administered and anonymous questionnaire was post on a survey platform named Questionnaire Star, and the generated link was distributed through WeChat alumni groups to graduated students by each university. Each WeChat account corresponded to a questionnaire qualification in case of repeated answering. In the present study, only necessary personal information was collected to ensure anonymity. The research protocol was approved by the Ethics Committee of the hospital of stomatology, Zhejiang Chinese Medical University (#201900516).

### Measurements

The questionnaire included 3 parts, socio-demographic characteristics (e.g., gender, age, living place, education level, clinical department, annual household income), turnover behavior and intention, and concerns about work-related factors. The United States Dollar (USD) was used to internationalize the currency description. The calculations utilized an average exchange rate of 1 USD = 7.07 CNY (Chinese Yuan) during the survey period.

Turnover behavior was judged by 2 questions “Have you ever been a medical staff” and “What’s your current occupation”. If the respondent used to be a medical staff but is not now, it means that the respondent has changed career. For the respondents who stick to the posts, a further question “How often you generate the thoughts of changing career” with a three-point scale was set to measure the degree of turnover intention. “0” means having no turnover intention, while “1” or “2” means having a little or high turnover intention. Concerns about work-related factors including salary, workload, occupational exposure, and doctor-patient relationship were surveyed. Question like “Do you think the salary need to be improved” was set to measure the degree of concern, ranging from 0 (no), 1 (neutral), and 2 (yes).

### Statistical analyses

The reliability and validity of the questionnaire were verified with Cronbach’s alpha coefficient and Kaiser-Meyer-Olkin (KMO) value, which was 0.71 and 0.73 respectively. Bartlett’s sphericity test result (x^2^ = 1185.197, *P* < 0.001) showed that the data were suitable for factor analysis [26].

In descriptive analyses, numbers (N) and percentages (%) were used for all variables. Chi-square tests were used to examine differences in turnover behavior and intention rates between dentists and medical doctors. The Wilcoxon Mann-Whitney test was used to examine different degree in turnover intention and concerns about work-related factors between dentists and medical doctors. Chi-square test was applied to find the potential correlated factors (*P* < 0.200), and those variables were further included in binary logistic regression models using forward stepwise method (lever for selection: *P <* 0.050). Odds ratio (OR) and 95% confidence interval (CI) were calculated to examine the association of the selected variables with turnover behavior and intention. In recent decades, gender-related issues have received much attention in the medical field [27, 28]. Therefore, in the present study, if gender was found to be associated with dentists’ or medical doctors’ turnover status, then the gender differences in the turnover status with different sociodemographic characteristics were further tested by binary logistic regression analysis. Data were analyzed using SPSS (IBM SPSS Statistics 25, IBM) and statistical significance was determined at *P* < 0.050.

## Results

**Fig. 1:**
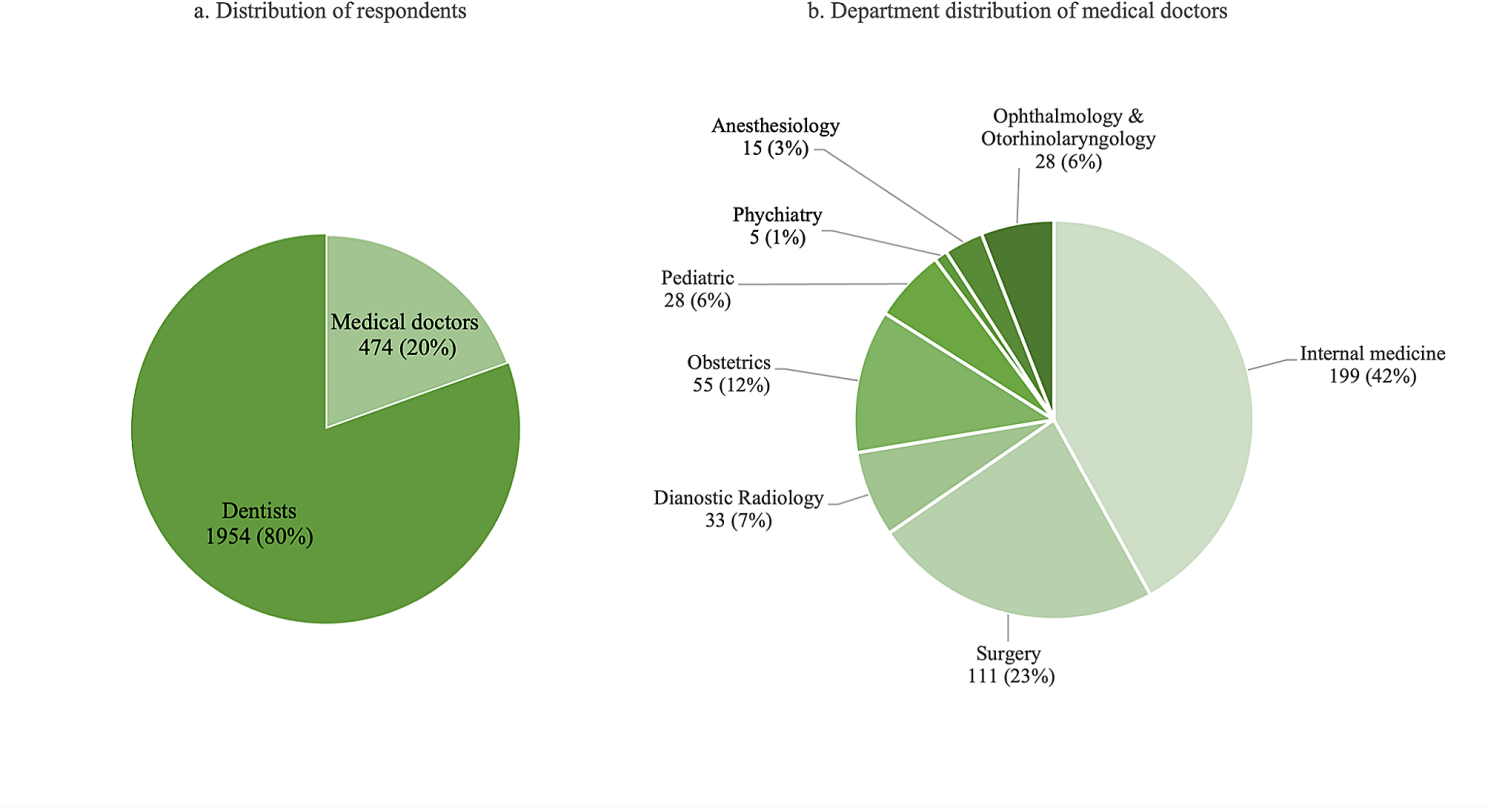
Distribution of the respondents in the present study (*N* = 2428)

A total of 3742 questionnaires were received, and after eliminating the questionnaires with logical errors and basic information missing, 2428 eligible questionnaires were used for the further analysis (effective response rate: 64.89%). Out of the 2428 respondents, the majority were dentists, and medical doctors were from 8 different departments (shown in Fig. 1). In the present study, some participants had junior college education. This might be a unique path to acquiring medical qualification in China. Person with a junior college degree can first work as dental/medical assistant for 2 years, and then applies for the qualification of dentist/medical doctor.

As shown in Table 1, the general turnover rate of respondents was 3.67%. Among the respondents who stuck to the posts, 55.45% had turnover intentions, but most of them did not have strong intentions. Dentists had significantly lower rates of both turnover behavior and intention than those of medical doctors (*P* < 0.001), and the degree of dentists’ turnover intention was also significantly lower than that of medical doctors’ (*P* < 0.001). For both dentists and medical doctors, the order of the degree of concerns of 4 working-related factors was consistent, that was salary, workload, strained doctor-patient relationship, and occupational exposure in descending order. However, there were significant differences in the degree of their concerns about different factors. Compared with medical doctors, dentists were more worried about occupational exposure and less concerned about workload, salary and strained doctor-patient relationship (*P* < 0.010, shown in Table 1).

**Table 1:** Turnover behavior/intention of medical doctors and dentists and their concerns about work-related factors (*N* = 2428)

	Overall (n, %)	Medical doctors (n, %)	Dentists (n, %)	***P*** value
Turnover behavior^†^				< 0.001***
Left the posts	89 (3.67)	33 (6.96)	56 (2.87)	
Stuck to the posts	2339 (96.33)	441 (93.04)	1898 (97.13)	
Turnover intention^†‡^ (Doctors who stuck to the posts)				
No	1042 (44.55)	127 (28.80)	915 (48.21)	< 0.001***
Yes	1297 (55.45)	314 (71.20)	983 (51.79)	
A little	1086 (83.73)	229 (72.93)	857 (87.18)	< 0.001***
High	211 (16.27)	85 (27.07)	126 (12.82)	
Concern about workload^‡^				< 0.001***
No	32 (1.32)	4 (0.84)	28 (1.43)	
Neutral	407 (16.76)	27 (5.70)	380 (19.45)	
Yes	1989 (81.92)	443 (93.46)	1546 (79.12)	
Concern about salary^‡^				< 0.004**
No	12 (0.49)	2 (0.42)	10 (0.51)	
Neutral	179 (7.37)	20 (4.22)	159 (8.14)	
Yes	2237 (92.13)	452 (95.36)	1785 (91.35)	
Concern about occupational exposure^‡^				< 0.001***
No	31 (1.28)	11 (2.32)	20 (1.02)	
Neutral	767 (31.59)	193 (40.72)	574 (29.38)	
Yes	1630 (67.13)	270 (56.96)	1360 (69.60)	
Concern about strained doctor-patient relationship^‡^				< 0.001***
No	18 (0.74)	2 (0.42)	16 (0.82)	
Neutral	474 (19.52)	65 (13.71)	409 (20.93)	
Yes	1936 (79.74)	407 (85.87)	1529 (78.25)	

†: Chi-square test was used; ‡: Wilcoxon Manny-Whitney test was used; †‡: Both Chi-square test and Wilcoxon Manny-Whitney test were used

*P < 0.050; **P < 0.010; ***P < 0.001

According to Chi-square tests, education level and concern about salary for both medical doctors and dentists, and place of residence and concern about occupational exposure for dentists, were found as the potential correlated factors of turnover behavior (*P* < 0.200, shown in Table 2). Then concern about salary for medical doctors, and place of residence and concern about occupational exposure for dentists, failed to enter the forward stepwise binary regression model (*P >* 0.050).

**Table 2:** Relationship of turnover behavior and the selected variables analyzed by chi-square tests

	Medical doctors (n,%)				Dentists (n,%)			
	Stuck to the posts				Stuck to the posts			
	Yes (*n* = 441)	No (*n* = 33)	X^2^	***P*** value	Yes (*n* = 1898)	No (*n* = 56)	X^2^	***P*** value
Gender			1.511	0.219			0.730	0.393
Male	262 (59.41)	17 (51.52)			740 (38.99)	25 (44.64)		
Female	179 (40.59)	16 (48.48)			1158 (61.01)	31 (55.35)		
Age (years)			0.210	0.900			0.802	0.670
19–25	16 (3.63)	1 (3.03)			239 (12.59)	7 (12.50)		
26–40	263 (59.64)	21 (63.64)			1149 (60.54)	31 (55.36)		
41–60	162 (36.73)	11 (33.33)			510 (26.87)	18 (32.14)		
Place of residence			0.415	0.715			3.571	0.154^§^
Countryside	5 (1.13)	0 (0.00)			53 (2.79)	4 (7.14)		
Town	54 (12.25)	5 (15.15)			165 (8.69)	5 (8.93)		
City	382 (86.62)	28 (84.85)			1680 (88.51)	47 (83.93)		
Education level			7.187	0.034^§^			24.687	< 0.001^§^
Junior college	2 (0.45)	2 (6.06)			36 (1.90)	7 (12.50)		
Bachelor’s	260 (58.96)	20 (60.61)			1231 (64.86)	28 (50.00)		
Master’s degree or above	179 (40.59)	11 (33.33)			631 (33.25)	21 (37.50)		
Concern about workload			2.602	0.335			2.301	0.286
No	3 (0.68)	1 (3.03)			26 (1.37)	2 (3.57)		
Neutral	26 (5.90)	1 (3.03)			369 (19.44)	11 (19.64)		
Yes	412 (93.42)	31 (93.94)			1503 (79.19)	43 (76.79)		
Concern about salary			4.990	0.125^§^			11.336	0.004^§^
No	1 (0.23)	1 (3.03)			8 (0.42)	2 (3.57)		
Neutral	18 (4.08)	2 (6.06)			150 (7.90)	9 (16.07)		
Yes	422 (95.69)	30 (90.91)			1740 (91.68)	45 (80.36)		
Concern about occupational exposure			0.554	0.705			3.814	0.141^§^
No	10 (2.27)	1 (3.03)			18 (0.95)	2 (3.57)		
Neutral	179 (40.59)	14 (42.42)			560 (29.50)	14 (25.00)		
Yes	252 (57.14)	18 (54.55)			1320 (69.55)	40 (71.43)		
Concern about strained doctor-patient relationship			1.277	0.510			1.597	0.409
No	2 (0.45)	0 (0.00)			15 (0.79)	1 (1.79)		
Neutral	59 (13.38)	6 (18.18)			399 (21.02)	10 (17.86)		
Yes	380 (86.17)	27 (81.82)			1484 (78.19)	45 (80.36)		

^§^*P* < 0.200

Variables (*P* < 0.200 in Chi-square tests) were incorporated into binary logistic regression models

The results of binary regression analysis are listed in Table 3. Compared with respondents with junior college education level, the turnover rate of other respondents with higher educational degrees were lower [dentists: bachelor’s degree (OR = 0.12, 95%CI = 0.05–0.31, master’s degree or above (OR = 0.19, 95%CI = 0.08–0.50); medical doctors: bachelor’s degree (OR = 0.08, 95%CI = 0.01–0.58), master’s degree or above (OR = 0.06, 95%CI = 0.01–0.48)]. Meanwhile, dentists who had concern about salary were less likely to change career than those who had no concern (OR = 0.11, 95%CI = 0.02–0.59). As gender was not identified as a correlated factor in the turnover behavior of both dentists and medical doctors, no additional statistical analyses were performed.

**Table 3:** Factors correlated to turnover behavior tested by binary logistic regression

	Medical doctors		Dentists	
Variables	OR (95% CI)	*P* value	OR (95% CI)	***P*** value
Education level				
Junior college	1		1	
bachelor’s	0.08 (0.01, 0.58)	0.012*	0.12 (0.05, 0.31)	< 0.001***
Master’s degree or above	0.06 (0.01, 0.48)	0.008**	0.19 (0.08, 0.50)	0.002**
Concern about salary	-	-		
No			1	
Neutral			0.26 (0.04, 1.55)	0.139
Yes			0.11 (0.02, 0.59)	0.010*

Some of the variables were eliminated automatically during analyzed by forward stepwise method (*P* > 0.05)

*P < 0.050; **P < 0.010; ***P < 0.001

According to Chi-square tests, age, annual household income, concerns about workload, salary, and strained doctor-patient relationship for both medical doctors and dentists, educational level for medical doctors, and gender, place of residence and concern about occupational exposure for dentists, were found as the potential factors correlated with turnover intention (*P* < 0.200, shown in Table 4). Then for medical doctors, annual household income and educational level failed to enter the forward stepwise binary regression model (*P* > 0.050). Meanwhile, for dentists, place of residence, concerns about salary and occupational exposure failed to enter the model (*P* > 0.050).

**Table 4:** Relationship of turnover intention and the selected variables analyzed by Chi-square tests

	Medical doctors (n,%)				Dentists (n,%)			
	Have turnover intention				Have turnover intention			
	No (*n* = 127)	Yes (*n* = 314)	X^2^	***P*** value	No (*n* = 915)	Yes (*n* = 983)	X^2^	***P*** value
Gender			0.110	0.740			15.099	< 0.001^§^
Male	50 (39.37)	129 (41.08)			398 (43.50)	342 (34.79)		
Female	77 (60.63)	185 (58.92)			517 (56.50)	641 (65.21)		
Age (years)			27.602	< 0.001^§^			18.934	< 0.001^§^
19–25	1 (0.79)	15 (4.78)			102 (11.15)	137 (13.94)		
26–40	56 (44.09)	207 (65.92)			526 (57.49)	623 (63.38)		
41–60	70 (55.12)	92 (29.30)			287 (31.37)	223 (22.69)		
Place of residence			1.722	0.457			4.200	0.122^§^
Countryside	2 (1.57)	3 (0.96)			23 (2.51)	30 (3.05)		
Town	12 (9.45)	42 (13.38)			68 (7.43)	97 (9.87)		
City	113 (88.98)	269 (85.67)			824 (90.06)	856 (87.08)		
Education level			4.806	0.075^§^			3.121	0.210
Junior college	2 (1.57)	0 (0.00)			22 (2.40)	14 (1.42)		
Bachelor’s	70 (55.12)	190 (60.51)			582 (63.61)	649 (66.02)		
Master’s degree or above	55 (43.31)	124 (39.49)			311 (33.99)	320 (32.55)		
Annual household income (USD)			18.904	< 0.001^§^			22.201	< 0.001^§^
<14,142	10 (7.87)	61 (19.43)			173 (18.91)	238 (24.21)		
14,142 − 42,426	88 (69.29)	210 (66.88)			448 (48.96)	512 (52.09)		
42,427 − 70,711	17 (13.39)	36 (11.46)			175 (19.13)	151 (15.36)		
70,712 − 141,421	11 (8.66)	6 (1.91)			90 (9.84)	68 (6.92)		
>141,421	1 (0.79)	1 (0.32)			29 (3.17)	14 (1.42)		
Concern about workload			16.757	< 0.001^§^			42.054	< 0.001^§^
No	-	-			21 (2.30)	5 (0.51)		
Neutral^#^	18 (14.17)	11(3.50)			224 (24.48)	145 (14.75)		
Yes	109 (85.83)	303 (96.50)			670 (73.22)	833 (84.74)		
Concern about salary			11.432	0.001^§^			17.591	< 0.001^§^
No	-	-			7 (0.77)	1 (0.10)		
Neutral^#^	12 (9.45)	7 (2.23)			93 (10.16)	57 (5.80)		
Yes	115 (8.66)	307 (97.77)			815 (89.07)	925 (94.10)		
Concern about occupational exposure			0.112	0.738			22.348	< 0.001^§^
No	-	-			10 (1.09)	8 (0.81)		
Neutral^#^	56 (44.09)	133 (42.36)			316 (34.54)	244 (24.82)		
Yes	71 (55.91)	181 (57.64)			589 (64.37)	731 (74.36)		
Concern about strained doctor-patient relationship			25.056	< 0.001^§^			65.910	< 0.001^§^
No	-	-			13 (1.42)	2 (0.20)		
Neutral^#^	34 (26.77)	27 (8.60)			258 (28.20)	141 (14.34)		
Yes	93 (73.23)	287 (91.40)			644 (70.38)	840 (85.45)		

^#^“Neutral” concerns about job-related factors in the medical doctor group was the combination of degree “no” and “Neutral” due to limited response as “no”, which will disturb the data analysis

^§^*P* < 0.200

Variables (*P* < 0.200 in Chi-square tests) were incorporated into binary logistic regression models

The results of binary regression analysis are listed in Table 5. Age, concerns about workload and strained doctor-patient relationship were the 3 common correlated factors of turnover intention in both dentists and medical doctors. In addition, for dentists, the females were more likely to have turnover intention than the males (OR = 1.39, 95%CI = 1.15–1.68). The turnover intention of dentists with low annual household income (less than 14,142 USD) was higher than those with high annual household income, especially for those with more than 70,711 USD annual household income [70,712–14,1421 USD (OR = 0.62, 95%CI = 0.41–0.92); >14,1421 USD (OR = 0.38, 95%CI = 0.19–0.76)]. For medical doctors, besides the 3 common correlated factors, having a neutral or no concern about salary (OR = 0.28, 95%CI = 0.09–0.85) correlated with lower turnover intention than those with high concerns regarding their salary.

**Table 5:** Factors correlated to turnover intention by binary logistic regression

	Medical doctors		Dentists	
Variables	OR (95% CI)	***P*** value	OR (95% CI)	***P*** value
Gender	-	-		
Male			1	
Female			1.39 (1.15, 1.68)	0.001**
Age (years)				
19–25	12.01 (1.50, 95.93)	0.019**	1.87 (1.31, 2.68)	0.001**
26–40	2.95 (1.88, 4.64)	< 0.001***	1.52 (1.22, 1.90)	< 0.001***
41–60	1		1	
Annual household income (USD)	-	-		
<14,142			1	
14,142 − 42,426			0.85 (0.66, 1.10)	0.199
42,427 − 70,711			0.73 (0.53, 1.01)	0.055
70,712 − 141,421			0.62 (0.41, 0.92)	0.017*
>14,1421			0.38 (0.19, 0.76)	0.006**
Concern about workload				
No	-	-	0.22 (0.08, 0.62)	0.004**
Neutral^#^	0.40 (0.16, 0.99)	0.048*	0.51 (0.40, 0.66)	< 0.001***
Yes	1		1	
Concern about salary			-	-
No	-	-		
Neutral^#^	0.28 (0.09, 0.85)	0.024*		
Yes	1			
Concern about strained doctor-patient relationship				
No	-	-	0.17 (0.04, 0.78)	0.023*
Neutral^#^	0.32 (0.17, 0.60)	< 0.001***	0.37 (0.19, 0.72)	0.003**
Yes	1		1	

Some of the variables were eliminated automatically during analyzed by forward stepwise method (*P* > 0.05)

^#^Neutral concerns about work-related factors in the medical doctor group was the combination of degree “no” and “Neutral” due to limited response as “no”, which will disturb the data analysis

*P < 0.050; **P < 0.010; ***P < 0.001

Gender was found to be a correlated factor in the turnover intention of dentists, rather than medical doctors. Further statistical analysis verified the existence of gender differences in dentists’ turnover intentions with different sociodemographic characteristics (Table 6). Among dentists who aged 26–40 (OR = 1.42, 95%CI = 1.12–1.81), living in the cities (OR = 1.55, 95%CI = 1.27–1.88), with bachelor’s educational level (OR = 1.49, 95%CI = 1.19–1.87), or with annual household income of 14,142 − 42,426 USD (OR = 1.45, 95%CI = 1.11–1.89), the females had higher degree of turnover intention than the males.

**Table 6:** Gender differences in dentists’ turnover intentions with different sociodemographic characteristics analyzed by binary logistic regression

	Male	Female	
Variables		OR (95% CI)	***P*** value
Age (years)			
19–25	Ref.	1.64 (0.96, 2.81)	0.072
26–40	Ref.	1.42 (1.12, 1.81)	0.004**
41–60	Ref.	1.27 (0.89, 1.81)	0.182
Place of residence			
Countryside	Ref.	1.05 (0.35, 3.11)	0.933
Town	Ref.	0.86 (0.46, 1.62)	0.645
City	Ref.	1.55 (1.27, 1.88)	< 0.001***
Education level			
Junior college	Ref	2.54 (0.55, 11.77)	0.234
Bachelor’s	Ref.	1.49 (1.19, 1.87)	0.001**
Master’s degree or above	Ref.	1.38 (0.98, 1.93)	0.062
Annual household income (USD)			
<14,142	Ref.	1.34 (0.89, 2.01)	0.163
14,142 − 42,426	Ref.	1.45 (1.11, 1.89)	0.006**
42,427 − 70,711	Ref.	1.32 (0.85, 2.05)	0.216
70,712 − 141,421	Ref.	1.11 (0.59,2.08)	0.748
>14,1421	Ref.	2.53 (0.69, 9.36)	0.163

*P < 0.050; **P < 0.010; ***P < 0.001

## Discussion

In this study, the turnover situation of both dentists and medical doctors in other clinical fields in China was investigated. The general turnover rate of all respondents was as low as 3.67%, but the turnover intention rate, 55.45%, was obviously higher than a previous study in China (20.5%) [8]. The difference may be due to different survey populations. In the previous study, the respondents all worked in tertiary hospitals, and all had a bachelor’s degree or above [8]. At the same time, as the top-ranked hospitals in China, the higher reputation of tertiary hospitals helps retain doctors [29].

In the present study, the turnover status of Chinese dentists was found to be more optimistic than medical doctors regarding both turnover behavior and intention rates. Dentists and medical doctors were consistent in their ranking of the 4 work-related factors in this study. Agreed with previous studies, salary was the most concerning factor for both dentists and medical doctors [30, 31]. Nevertheless, the extent of their apprehension regarding various factors exhibited noteworthy disparities. Dentists were more concerned than medical doctors about occupational exposure. This may be because many clinical procedures in dentistry generate aerosols, which pose a potential risk of infection transmission [32]. Dentists were significantly less worried about strained doctor-patient relationship than medical doctors due to the less workplace violence [24]. Meanwhile, Chinese medical doctors were more concerned about salary and workload than dentists, and the reason should be clarified in the future.

The present study found that the education level of medical doctors and dentists was negatively related to their turnover behavior, which was consistent with previous finding [33]. It may be due to the fact that perception of an imbalance in effort-reward being more common among doctors with lower levels of education [6, 34]. However, Ran et al. [35] found that the education level of primary healthcare staff in China was positively correlated with turnover intention. This may be due to differences in the survey populations. Most of the respondents in this study lived in cities, while the respondents in the previous study mainly lived in towns or countryside, and the highly educated doctors may have stronger willing to leave and to find better job opportunities than others [35]. Besides education level, salary concern was negatively associated with dentists’ turnover behavior. It indicated that being concerned about salary did not mean that dentists were dissatisfied with salary, but that dentists had high expectations for work and remuneration.

Age was a common factor related to the turnover intention of both dentists and medical doctors in the present study. Previous studies also found that younger doctors had higher turnover intention than the elders in China [30, 36]. This may be related to job satisfaction [36]. Being elder generally means more experience, so experienced dentists experience less occupational stress and have greater job satisfaction than younger dentists with less experience [37]. Therefore, helping young dentists and medical doctors improve their professional skills may be beneficial to relieving their work pressure, improving job satisfaction, and thereby reducing their turnover intention. As for the work-related factors, concerns about doctor-patient relationship and workload were the common factors correlated with turnover intention of both Chinese medical doctors and dentists. In many countries, doctors’ complaint of being overburdened with work, which can further lead to emotional exhaustion and even turnover intentions [6, 9, 10]. At the same time, strained doctor-patient relationships were a global problem. According to a survey conducted in Guangdong, China, it was reported that almost half of the doctors experienced at least one medical dispute over the previous 12 months, and 87.6% of the doctors deemed doctor-patient relationship as tense or very tense [38]. Some studies found that more than 60% doctors experienced work violence at least once a year [12–14]. The workplace violence may lead to career disappointment and in turn positively related to turnover intention for health workers [39].

In addition to common factors, there were some distinct factors correlated with dentists’ and medical doctors’ turnover intention. Previous studies usually focus on the correlation between personal income and turnover intention [8, 34]. This study included two variables, annual household income and concern about salary. The results showed that for medical doctors, turnover intention was positive correlated to concern about salary and has nothing to do with family income, while for dentists, the results were opposite. Agreed with previous findings in both developed and developing countries, medical doctors often had pessimistic attitude toward salary [7, 11]. It may not be due to the salary itself or poor economic conditions, but rather because of the effort-reward imbalance. A meta-analysis showed that the prevalence of effort-reward imbalance among doctors worldwide was 40.02% and was related to turnover intention [6, 40]. Therefore, medical doctors’ workload must be taken into account when formulating a compensation system for them. Dentists’ turnover intentions were not related to their concern about salary but were negatively related to annual household income. This implied that the effort-reward of Chinese dentists may be balanced. When the overall economic situation of the family was good, especially when it was higher than the average in Chinese cities (70,712 USD in 2020) [41], dentists had lower intention to leave.

In previous studies, the correlation between gender and doctors’ turnover intention has been controversial [8, 36, 42]. In this study, the correlation between gender and respondents’ turnover status was only found for dentists’ turnover intentions. Compared with the male dentists aged 26–40, the females had a higher intention to leave. This may be related to the fact that female dentists in this age group struggle to balance their career with the demands of motherhood, and their job satisfaction was lower than that of the males [28, 37]. At the same time, female dentists with a bachelor’s degree or living in cities had higher turnover intentions than the males. With the advancement of education, the number of undergraduates continued to increase, and undergraduates were more inclined to work in cities, which increased the competitive pressure for city employment [43]. Researchers have found that, among individuals with the same level of education, males were more likely to secure employment, receive higher salaries, and experience greater promotional opportunities compared to their female counterparts. This disparity contributed to lower job identity and a heightened likelihood of turnover intentions among females [44, 45]. When the family income level was low, the turnover intention of female dentists was significantly higher than that of the males. A study showed that fewer female than male dentists were the main source of household income [28]. Given that dentists’ concern about salary showed no significant correlation with their turnover intention, one can infer that female dentists generally found satisfaction in their job and only contemplated changing careers when faced with deteriorating family economic circumstances as a means to enhance their financial situation.

Similar to their Chinese counterparts, doctors in many other countries encounter significant work challenges as well. However, variations in culture, medical insurance systems, and resource allocation mean that the challenges faced by doctors in different countries can be diverse [46, 47]. Therefore, it’s important to interpret the results of this study with caution. China, with its large population, experiences a noticeable shortage of medical resources across various fields. This study, the first of its kind to examine the turnover status and influencing factors among dentists in China, holds significant value. It provides essential insights for monitoring future changes in dental practitioners and developing effective policies to retain dentists.

The present study had some limitations. First, unbalanced distribution of medical doctors among departments may have an impact on the results. Second, this was a cross-sectional study and cannot determine causal relationships between factors and turnover behavior and intention. Longitudinal studies are needed to confirm causal relationships, and to propose and verify the validity of related measures regarding doctors’ retention.

## Conclusion

Low turnover rate but high turnover intention rate was the current status of Chinese doctors’ employment. Turnover behavior and intention were more optimistic among dentists than medical doctors. Educational level was the common factor correlated with turnover behavior, and age, concerns about workload and doctor-patient relationship were the common factors correlated with turnover intention in both dentists and medical doctors in China. The distinct factors that influence the turnover behavior and intention of dentists and medical doctors existed. Therefore, in addition to generic measures, individualized measures were necessary for retaining dentists and medical doctors.

## Data Availability

Data are available upon reasonable request. Data can be available by contacting the corresponding author.

## Abbreviations

WHO: World Health Organization
USD: United States Dollars
CNY: Chinese Yuan
OR: Odds ratio
CI: Confidence Interval
